# Project Description: DNA Barcodes of Bird Species  in the National Museum of Natural History, Smithsonian Institution, USA

**DOI:** 10.3897/zookeys.152.2473

**Published:** 2011-12-08

**Authors:** David E. Schindel, Mark Y. Stoeckle, Chris Milensky, Michael Trizna, Brian Schmidt, Christina Gebhard, Gary Graves

**Affiliations:** 1 Consortium for the Barcode of Life, MRC-105, National Museum of Natural History, Smithsonian Institution, P. O. Box 37012, Washington, D.C. 20013-7012 USA; 2 Program for the Human Environment, Rockefeller University, New York, USA; 3 Department of Vertebrate Zoology, MRC-116, National Museum of Natural History, Smithsonian Institution, P. O. Box 37012, Washington, D.C. 20013-7012 USA; 4 Center for Macroecology, Evolution and Climate, University of Copenhagen, DK-2100 Copenhagen, Denmark

**Keywords:** DNA barcoding, GenBank, BOLD, genomics

## Abstract

The Division of Birds, National Museum of Natural History, Smithsonian Institution in Washington, DC, has obtained and released DNA barcodes for 2808 frozen tissue samples. Of the 1,403 species represented by these samples, 1,147 species have not been barcoded previously. This data release increases the number of bird species with standard barcodes by 91%. These records meet the data standard of the Consortium for the Barcode of Life and they have the reserved keyword BARCODE in GenBank. The data are now available on GenBank and the Barcode of Life Data Systems.

## Introduction

The Division of Birds, National Museum of Natural History of the Smithsonian Institution (USNM), has released approximately 2800 DNA barcode data records into the public domain through GenBank and the Barcode of Life Data Systems (BOLD). These records were derived from the Division’s extensive collection of frozen tissues that are linked to voucher specimens in the Museum. The data adhere to the DNA barcode data standard (Consortium for the Barcode of Life 2005) and accordingly they have been labeled by GenBank with the reserved keyword ‘BARCODE’. This new public dataset adds 1,147 newly barcoded species to the 1,259 species in GenBank that meet the BARCODE data standard. This increase of 91% in the DNA reference library for birds serves as a model for how frozen tissue collections in major biorepositories can be digitized through barcoding and made more accessible to the research community.

This ‘Project Description’ has been submitted as part of a policy of rapid data release for genomic data known as the Fort Lauderdale Principles (Wellcome Trust 2003). These principles described a system of shared responsibility that would be needed to create incentives to construct, publish and use large public genome datasets such as that of the Human Genome Project. The Principles have not been implemented or even discussed to any extent in the taxonomic community. Stated briefly, the Principles:

•	Urge funding agencies to require the early and rapid release of large genomic datasets that represent research infrastructure with significant potential for use by the research community beyond the data producers;

•	Encourage data producers to publish Project Descriptions such as this one to state their intended use of a newly released dataset within a stated, reasonable period of time;

•	Propose that researchers should be expected to refrain from using the data for purposes and interval stated in the Project Description, but should be free to use the data for other applications with proper citation of the Project Description or other references to the dataset.

A full description of the dataset is in preparation with the goal of publication as a 'data release paper' in ZooKeys before June 2012, in accordance with guidelines issued by ZooKeys (Penev et al. 2011) and CBOL (Consortium for the Barcode of Life 2008). The data release paper will present summary statistics on the variability within and among species of the DNA barcode region (648 nucleotides representing approximately the 5’ half of the mitochondrial cytochrome *c* oxidase I gene). The paper will describe the geographic range covered by samples, numbers of samples analyzed per species, and the methods used in the cryo-collection, laboratory, and post-sequencing data processing. The impact of barcoding on collection management and curation will also be addressed in the data release paper.

The data release paper will also discuss the relationship between clusters based on barcode data variability and taxonomic names attached to the voucher specimens from which the DNA barcodes were derived. The taxonomic identifications in the GenBank records have undergone screening relative to each other and there are some uncertainties associated with some species-level determinations. These will be investigated more carefully by re-examining voucher specimens and analysis of the barcode sequences relative to other public barcode records. All species determinations will be resolved by the time of publication of the full data release paper.

## Data resources

Data are deposited in GenBank under accession numbers JQ173884-JQ176686 (http://www.ncbi.nlm.nih.gov/nuccore?term=JQ173884:JQ176686[accn]). The full dataset is also available on BOLD at http://www.barcodinglife.org as project name ‘USNMY’ under ‘Published Projects’.

## Contents of the dataset

The dataset represents samples from 27 countries (Argentina, Australia, Botswana, Brazil, Gabon, Greece, Guyana, Iceland, Johnston Atoll, Mariana Islands, Mexico, Mongolia, Myanmar, Pakistan, Panama, Papua New Guinea, Philippines, Puerto Rico, Russia, South Korea, St. Vincent, Swaziland, Sweden, United Kingdom, United States, Uruguay, and the former Soviet Union).

Each GenBank record in the dataset carries the BARCODE keyword that indicates compliance with CBOL’s barcode data standard. Accordingly, each record includes the following data elements required by the standard:

•	The name of the approved BARCODE region (COI in this case).

•	A species level identification. All names can be found in the Integrated Taxonomic Information System (ITIS 2011) or Clements (2007).

•	A structured identifier of the voucher specimen using the Darwin Core triplet consisting of institutional acronym, collection code, and specimen ID number.

•	Country of origin.

•	Forward and reverse primer sequences.

•	A DNA sequence based on forward and reverse sequencing reactions with at least 75% coverage of the standard barcode region as specified in

In addition, many records include the following data fields that are strongly recommended by the standard:

•	Latitude and longitude of collecting locality

•	Date of collection

•	Name of collector

•	Name of identifier

## Use of early release data

The authors invite the research community to examine and analyze the data in their current form with the following understandings:

•	As with all data released on GenBank, the National Center for Biotechnology Information places no restriction on their use or distribution.

•	The authors intend to publish a descriptive paper summarizing the dataset and its implications for bird barcoding and any taxonomic issues arising from the data. Publication of this data release paper is anticipated by 1 June 2012. In accordance with the Fort Lauderdale Principles (Welcome Trust 2011), the authors ask the community to respect our intent to publish on these topics and not to submit manuscripts for this purpose based on this dataset.

•	Use of this dataset for purposes other than those described above are welcome and encouraged, contingent on proper citation of this publication.

•	The authors invite members of the community to examine the data and test their accuracy relative to other datasets. We welcome your comments, suggestions and corrections. BOLD 3.0 includes the capability to submit annotations to data submitters and we encourage readers to use this new system to submit observations on this dataset.

•	The species determinations are not yet final. Some of the species identification may be change by the time of publication of the data release paper (anticipated by 1 June 2012).
